# Variations of Bioactive Phytochemicals and Antioxidant Capacity of Navel Orange Peel in Response to Different Drying Methods

**DOI:** 10.3390/antiox11081543

**Published:** 2022-08-09

**Authors:** Chunling Lai, Yan Liang, Linyan Zhang, Jiangjiang Huang, Kumaravel Kaliaperumal, Yueming Jiang, Jun Zhang

**Affiliations:** 1 National Engineering Research Centre of Navel Orange, Gannan Normal University, Ganzhou 341000, China; 2 Key Laboratory of Plant Resources Conservation and Sustainable Utilization, South China Botanical Garden, Chinese Academy of Sciences, Guangzhou 510650, China

**Keywords:** drying methods, orange peel, chemical composition, antioxidant activity

## Abstract

The effects of five different drying methods, namely, freeze drying (FD), shade drying (SD), hot-air oven drying at 50 °C (OD50), 70 °C (OD70), and microwave drying (MD) on the bioactive phytochemicals and antioxidant capacity of navel orange peel were assessed and comprehensively discussed in detail. Compared with other drying methods, MD-treated peel contained the lowest total phenolic content (TPC) and total flavonoid content (TFC). The peel subjected to OD70 treatment was superior in TPC relative to other treatments and the highest TFC was found in the peels treated with FD. HPLC analysis identified thirteen flavonoids involving three flavanone glycosides (FGs) and ten polymethoxyflavones (PMFs) in navel orange peel and revealed that PMFs in peel were stable under all these drying methods, whereas the three major FGs (narirutin, hesperidin, and didymin) in peel significantly degraded in response to MD treatment. The peels subjected to OD50/OD70 treatments had the most potent antioxidant capacity when compared to other drying methods. Furthermore, Pearson’s correlation analysis was performed. The results revealed here allow us to recommend the use of OD50 or OD70 for the drying of orange peel, both of which help the maintenance of bioactive compounds in the peel and improve its antioxidant capacity.

## 1. Introduction

Navel orange, one of the main varieties of citrus fruits, is widely grown in Ganzhou of Jiangxi Province, China. In 2021, the total planting area and the annual output of navel orange in Ganzhou reached over 100 thousand hectares, and over 1 million tons, respectively [1]. With the consumption of orange flesh, large quantities of peels were discarded, which caused the waste of resources and environmental pollution, thus raising concerns about the value-added utilization of this abundant resource [2]. Citrus peels contain large amounts of biologically active compounds, mainly phenolic acids and flavonoids, which exhibit pronounced antioxidant, neuroprotective, anti-inflammatory, antiproliferation, antiallergy, and antiviral properties [3,4,5]. Therefore, orange peel could be considered a valuable source of bioactive substances which may function as important ingredients in the production of cosmetics, pharmaceuticals, and nutraceuticals [6,7]. Fresh orange peel with a moisture content of over 70% is difficult to preserve due to the deterioration caused by microbes or enzymes [4]. Drying is one of the extensively used methods to extend the storage life of orange peel by removing water to inhibit spoilage as well as reduce the activity of enzymes [4,7]. In general practice, the drying process of the citrus peel should be conducted before carrying out further procedures, such as storage, transportation and extraction of bioactive components.

Several drying techniques have been applied to the dehydration of citrus byproducts, involving shade drying, sun drying, far-infrared radiation drying, hot-air drying, freezing drying, microwave drying, etc. [3,4,8]. Shade drying usually takes a long time and is generally applied to some traditional Chinese medicines containing volatile oils or mucus [3]. Freeze drying can prevent the degradation of heat or oxygen-sensitive bioactive compounds, but requires high energy consumption and lengthy processing time, making it much more suitable for some high-value products [8]. In contrast, hot-air drying is a relatively inexpensive and user-friendly method [9]. Microwave drying has been reported to efficiently transfer energy for the removal of moisture, thus decreasing the drying time and preserving the quality of the product, whereas it is hard to control the temperature to have a homogeneous treatment [10]. The effects of different drying methods on the chemical and biological properties of citrus products have been reported previously, but with some controversial results, suggesting that more influential factors such as citrus cultivar, the parameter of drying method, and extracting method, might influence the results as well. For example, the freeze-drying method resulted in the highest total phenolic content (TPC) and antioxidant effects in the immature citrus fruits of four citrus species (Ponkan, Gaocheng, Foyu, and Huyou), compared to the hot-air and sun drying methods [3]. However, Papoutsis et al. reported that the lemon pomace dried by hot air at 110 °C had the highest TPC, whereas the freeze-dried one had the lowest [8]. On the other hand, Ledesma-Escobar et al. reported that the lemon dried by freeze-drying had higher TPC than that dried by hot-air drying at 45 °C [11]. The total polyphenols, flavonoids, ascorbic acid, and antioxidant capacity of orange peel markedly decreased after drying processing [7]. In other studies, the highest antioxidant capacity for orange peel was obtained when the peel was dried in a hot-air oven at 60 °C [9], and the microwave drying at 450W significantly improved the extractible amounts of phenolics compared to the fresh orange peel [10]. Therefore, the selection of suitable drying methods for orange peel is challengeable and it plays an important role in realizing the optimal valorization of orange peel. To the best of our knowledge, the research about the drying methods of peel from navel orange planted in Ganzhou of China is largely unavailable yet.

Herein, the effects of five different drying methods, namely, freeze drying (FD), shade drying (SD), hot-air oven drying at 50 °C (OD50), 70 °C (OD70), and microwave drying (MD) on the chemical composition and antioxidant capacity of navel orange peel were evaluated for the first time. In the present study, the chemical compositions involving TPC, total flavonoid content (TFC) and thirteen individual flavonoids were comparatively analyzed, and three antioxidant assays as 2,2-diphenyl-1-picrylhydrazyl free radical (DPPH), 2,2′-azinobis-(3-ethylbenzthiazoline-6-sulphonate) free radical (ABTS), and ferric reducing antioxidant power (FRAP) assays were employed together for the antioxidant tests. Moreover, Pearson’s correlation analysis was adopted to examine the relationships among all variables tested. The present results might provide useful information for the drying processing of orange peel, and contribute to the value-added utilization of this abundant side-product.

## 2. Materials and Methods

### 2.1. Chemicals

2,4,6-Tris(2-pyridyl)-s-triazine (TPTZ), 2,2-diphenyl-1-picryhydrazyl (DPPH, 97%), 2,2-azino-bis(3-ethylbenzothiazoline-6-sulfonic acid) diammonium salt (ABTS, 98%) and Folin–Ciocalteu reagent (1 M) were purchased from Solarbio Science and Technology Co., Ltd. (Beijing, China). Methanol, 95% ethanol, petroleum ether, ethyl acetate, dichloromethane, and N, N-Dimethylformamide (DMF) were of analytical grade and purchased from Damao Chemical Reagent Factory (Tianjin, China). The flavonoids involving narirutin, hesperidin, didymin, isosinensetin, 3,3′,4′,5,7,8-hexamethoxyflavone, sinensetin, 4′,5,7,8-tetramethoxyflavone, 3,3′,4′,5,6,7-hexamethoxyflavone, nobiletin, 4′,5,6,7-tetramethoxyflavone, 3,3′,4′,5,6,7,8-heptamethoxyflavone, 5-hydroxy-6,7,3′,4′-tetramethoxyflavone, and tangeretin were isolated from the orange peel by authors according to chromatographic methods [2] and each had a purity of >95% (data shown in the supplement material). Acetonitrile (ACN) was of high-performance liquid chromatography (HPLC) grade (Anaqua Chemicals Supply, Houston, TX, USA). De-ionized water used for chromatography was obtained from a Milli-Q Gradient A10 system (Millipore, Billerica, MA, USA). All other chemicals were of analytical grade and purchased from Sigma-Aldrich (Shanghai, China).

### 2.2. Plant Materials

Newhall navel oranges (50 kg) at a commercial mature stage were harvested on 15 December 2020 from an orchard located in Ganzhou (23.1291° N, 113.2644° E) of Jiangxi Province, China. The orange trees were planted in red-yellow loam soil (pH 5.39 ± 0.3) and spaced at 4 m and 3 m between and along the rows, respectively. The freshly harvested fruits were transferred to the laboratory, washed and squeezed immediately. The peels were then collected from the pomace by hand and were cut into pieces of approximately 1 cm^2^ each. The pieces of peel were pooled together and stored at −80 °C in sealed plastic bags until drying experiments.

### 2.3. Drying of Orange Peel

Orange peels were subjected to five different drying methods termed FD, SD, OD50, OD70, and MD. For each treatment, 1 kg of orange peel was used. The detailed procedures were described as follows: (1) For FD, the pieces of peel were spread out on trays to form a thin layer of about 0.5 cm thickness. The peels on trays were pre-frozen in the refrigerator at −80 °C overnight. The frozen peels were then dried to a constant weight for 48 h by using a freeze dryer (FD8-6 P, SIM International Group, Newark, NJ, USA). (2) For SD, the pieces of peel were spread out on trays to form a thin layer having a thickness of about 0.5 cm. The trays were kept in a well-ventilated and shady area at ambient temperature (approximate temperature 5–20 °C) and 60–80% relative humidity for 14 days to achieve a constant weight. (3) For both OD50 and OD70, the peels were distributed on a stainless steel wire mesh to form a thin layer (thickness of about 0.5 cm) and dried in a hot-air oven (DHG-9240A, Jinghong Co., Shanghai, China) at 50 or 70 °C with 2 m/s air flow rate and 5–10% relative humidity for 12 h (OD50) or 8 h (OD70) to achieve a constant weight. (4) The MD treatments were performed in a domestic microwave oven equipped with a glass turntable (M1-L204A, Midea, Guangdong, China) with the following features: 220 V (voltage), 1150 W (input power), 700 W (output power) and 2450 MHz (operating frequency). The peels were distributed on glass dishes to form a single layer (thickness of about 0.5 cm). The dishes containing peels were then placed on the turntable of a microwave oven and dried at 600 W for 12 min to achieve a constant weight.

After drying, samples were kept in a desiccator overnight to allow a homogeneous distribution of moisture, and the final moisture content was 10 ± 0.5% wet basis. The dried peels were ground to a powder by using an electric grinder (QE-100, Zhejiang YiLi Tool Co., Ltd., Jiaxing, China), followed by sieving through a 40-mesh sieve. The powders were packed in plastic bags, labeled and stored at −80 °C until extraction. The experiments were performed in triplicate for each drying method.

### 2.4. Extraction of Dried Orange Peel

Maceration extraction at room temperature was employed to extract bioactive compounds from the dried orange peel. Briefly, dried peel powders (5 g) were added into a 250 mL conical bottle, followed by the addition of 50 mL of 95% ethanol. The conical bottle was placed on the bench for 24 h with occasional shaking. After that, the mixture was filtered with Whatman filter paper (No 1) and the residue was collected and repeatedly extracted four times as described above. All filtrates were combined and concentrated by a vacuum rotary evaporator at 35 °C to give a brown residue, which was subjected to lyophilization for 48 h by using a freeze dryer (FD8-6 P, SIM International Group, Newark, NJ, USA) and stored at −80 °C until further analysis. The experiments were performed in triplicate for each dried sample.

### 2.5. TPC Analysis

TPC was determined spectrophotometrically with the Folin–Ciocalteu reagent according to one of our previous reports [2]. Briefly, the extract solution in methanol (20 μL), distilled water (60 μL), and Folin–Ciocalteu reagent (15 μL, pre-diluted from 1 M to 0.5 M with water at a volume ratio of 1:1) were sequentially added to a 96-well plate and mixed well by smoothly shaking for 3 min. After 4 min of static incubation at room temperature, 75 μL Na_2_CO_3_ aqueous solution (2% *w/v*) was added, followed by slightly shaking for 3 min. The optical density was measured at 750 nm using a microplate reader (Tecan Spark 10M, Männedorf, Switzerland) after 15 min of static incubation at room temperature. The methanol was used as a blank control. TPC was calculated from a linear calibration curve (y = 0.0046x + 0.012, R^2^ = 0.9998, linear range from 6.25 to 100 mg/L) which was constructed by plotting the absorbance values against the concentrations of gallic acid, and expressed as micromole of gallic acid equivalent per gram dry weight of extract (μM GAE/g DW). Each measurement was conducted in triplicate.

### 2.6. TFC Analysis

TFC was estimated by the method described previously [2]. Briefly, 500 μL NaNO_2_ (5% *w/v*), 500 μL AlCl_3_ (10% *w/v*) and 500 μL NaOH (1.0 M) were sequentially added to a 10 mL volumetric flask containing 500 μL of extract solution in methanol at 0, 5 and 11 min, respectively. Each addition was followed by gentle shaking. Methanol was used as a blank control. The reaction mixture was kept at room temperature for 15 min with occasional shakings, distilled water was then added to achieve a final volume of 10 mL. The absorbance of the reaction mixture was measured at 415 nm by a UV-vis photospectrometer (Model 2450, Shimadzu Co., Ltd., Kyoto, Japan). TFC was calculated from a linear calibration curve (y = 0.0013x + 0.0031, R^2^ = 0.9996, linear range from 31.25 to 500 μg) which was constructed by plotting the absorbance values against the amounts of quercetin, and expressed as micromole of quercetin equivalent per gram dry weight of extract (μM QE/g DW). Each measurement was conducted in triplicate.

### 2.7. HPLC Analysis

The individual flavonoids were analyzed according to the method reported previously with some minor modifications [12]. The peel extract was dissolved in 5% DMF/methanol solution (*v/v*) to form a clear solution with a concentration of 25 mg/mL, which was then filtered through a 0.22 μm Millipore filter. The flavonoids of the extract were analyzed by Agilent 1200 HPLC system coupled with an XBridge-C18 reverse phase column (150 mm length × 4.6 mm id, 5.0 μm particle size) at 340 nm detection wavelength. The mobile phase consisted of acetonitrile (A) and water (B) with a gradient elution: 10–25% A (0–15 min), 25–35% A (15–25 min), 35–50% A (25–50 min), 50–90% A (50–60 min), 90% A (60–70 min), 90–10% A (70–75 min). The injection volume was 20 μL with a flow rate of 1.0 mL/min. The identification of individual flavonoids in the extract was achieved by comparisons of retention time and UV absorption pattern with those of standard compound, and the calibration equation was used to quantify the amount of each flavonoid with the result being expressed as μg flavonoid per mg of the dry weight of peel extract (μg/mg DW). The retention time, regression parameters, and linear range of standard compounds analyzed by HPLC were reported in Table S1 (Supplementary File). Each measurement was conducted in triplicate.

### 2.8. DPPH Scavenging Assay

DPPH assay was performed according to the method reported previously with some modifications [13]. Briefly, DPPH was freshly prepared in methanol at a concentration of 0.1 mM. The peel extract was dissolved in methanol and subjected to two-fold dilution with methanol to prepare desired solutions with concentrations ranging from 0.625 to 10 mg/mL. In total, 50 μL of each solution was placed into a 96-well plate, followed by the addition of a freshly prepared DPPH reagent (150 μL). The plate was incubated at 37 °C in dark for 20 min, then the absorption at 517 nm was detected by using a microplate reader (Tecan Spark 10M, Männedorf, Switzerland). Vitamin C (VC) was used as a positive control. The measurements were performed in triplicate with three replications. The inhibitory rate was calculated according to the formula: Inhibition (%) = [1 − (A_treated_ − A_blank_)/A_control_] × 100, where A_treated_ represents the average absorption of wells adding both DPPH and extract solution, A_blank_ is the average absorption of wells only adding extract solution, and A_control_ is the average absorption of wells only adding DPPH. The IC_50_ value represents the sample concentration scavenging 50% of the DPPH radical.

### 2.9. ABTS Scavenging Assay

ABTS radical scavenging ability was determined according to a previous method with some minor modifications [2]. The ABTS aqueous solution (7 mM) and ammonium persulfate aqueous solution (2.45 mM) were mixed at a volume ratio of 1:1 and reacted in dark at room temperature for 12 h to form a stable ABTS radical solution, which was then diluted with 70% ethanol/water to an absorbance of 0.70 ± 0.02 at 734 nm before use. The peel extract was dissolved and two-fold diluted with 70% ethanol/water to prepare test solutions. In total, 50 µL of each test solution was added to the wells of a 96-well plate, followed by the addition of freshly prepared ABTS radical solution (200 μL). A 70% ethanol/water and vitamin C mixture was employed as a blank and positive control, respectively. The plate was smoothly shaken for 10 min, then the absorbance was measured at 734 nm by using a microplate reader (Tecan Spark 10M, Männedorf, Switzerland). The scavenging capacity was calculated according to the formula: Inhibition (%) = [1 − (A_treated_−A_blank_)/A_control_] × 100, where the A_treated_ represented the average absorption of wells containing both ABTS and extract solution, A_blank_ was the average absorption of wells containing only extract solution, and A_control_ was the average absorption of wells containing only ABTS. The measurements were performed in triplicate with three replications. The IC_50_ value was expressed as the concentration scavenging 50% of ABTS radical.

### 2.10. FRAP Assay

FRAP assay was carried out according to a previous method with minor modifications [14]. FRAP solution was prepared by mixing TPTZ (10 mM in 40 mM HCl aqueous solution), acetate buffer (0.1 mM, pH 3.6), and ferric chloride (20 mM in 40 mM HCl aqueous solution) at a volume ratio of 1:10:1. The peel extract was dissolved and two-fold diluted with 70% ethanol/water to give test solutions. In total, 50 µL of each test solution was added to the wells of a 96-well plate, followed by the addition of a freshly prepared FRAP reagent (200 μL). A 70% ethanol/water and vitamin C mixture was used as the blank and positive control, respectively. The plate was smoothly shaken for 10 min, and the absorbance was then measured at 593 nm by a microplate reader (Tecan Spark 10M, Männedorf, Switzerland). The measurements were performed in triplicate with three replications. FRAP value was calculated from a linear calibration curve (y = 0.0345x − 0.0084, R^2^ = 0.9997, linear range from 1.56 to 50 mg/L) which was constructed by plotting the absorbance values against the concentrations of vitamin C (VC), and expressed as μg of VC equivalent antioxidant capacity per mg of the dry weight of the extract (μg VC/mg).

### 2.11. Statistical Analysis

Data were expressed as mean ± standard deviation (SD) of independent experiments performed in triplicate and were analyzed by using one-way analysis of variance (ANOVA) (*p* < 0.05) with SPSS 21 (IBM Corporation, Armonk, NY, USA). Pearson’s correlation analysis was performed using SPSS 21 (IBM Corporation, Armonk, NY, USA) and the heatmap was obtained by the software TBtools (version 1.0692).

## 3. Results and Discussion

### 3.1. Effect of Different Drying Methods on the Extracting Yield, TPC, and TFC

The maceration extraction at room temperature was used to extract the dried peel in the present study since it had a minimum side effect on the chemical components compared to other extraction methods employing heat or sonication treatments which might cause degradation or decomposition of compounds. Therefore, the present result might give convincing results reflecting the effect of only different drying methods without the influence of extraction processing. The extracting yield was shown in Table 1. The peel treated with MD had the best extracting yield (35.63%) when compared to other treatments, followed in sequence by FD (34.59%), OD50 (33.33%), OD70 (32.08%), and SD (32.44%) treatments. Similarly, a previous study has reported that freeze drying has an obvious advantage in extracting yield compared with the hot-air drying method [15]. The microwave radiation might make the fiber matrix become larger and looser and promote the formation of a more porous structure in the peel, thus facilitating the extraction with solvent, which might account for the highest extracting yield for MD treatment in the present study. This assumption was supported by previous studies indicating that the pore size of the citrus peel dried by the microwave method was greater than that of the citrus peel dried by the hot-air method [16] and that the fiber structure in hot-air dried shiitake mushrooms was arranged tightly while the microwave-dried samples demonstrated a clear porous structure [17].

The TPC in peel subjected to different drying methods was listed in the order OD70 > OD50 > SD > FD > MD. As shown in Table 1, the two highest TPC values were obtained both from the OD treatments, suggesting that the heat treatment might enhance the production of extractable phenolics in the peel. This suggestion was supported as well by the TPC results showing OD > SD > FD, where the drying temperature decreased in sequence. The phenolics in citrus were present in two forms—bound or free form—and the bound form was supposed to be liberated to the free form as the temperature increased [3]. The present result supported this assumption and suggested that the high drying temperature would liberate some bound phenolics to free form, thus resulting in more extractable phenolics in OD-treated peel than those in SD or FD-treated ones. Similarly, Papoutsis et al. reported that TPC was higher in lemon pomace dried by hot air than that dried by freeze drying and it increased as the drying temperature increased [8].

In addition, the TPC result in the present study could be attributed to the presence of polyphenol oxidase (PPO) in the peel as well. PPO is an enzyme responsible for the selective oxidation of polyphenols, and its activity tends to reduce as temperature increases [18]. In the present study, the PPO activity in OD70-treated peel should be lower than in other treatments since the drying temperature during OD70 processing is 70 °C, which might deactivate the enzymatic activity and cause less oxidative degradation of phenolics, thus resulting in the highest TPC value in the peel treated with OD70 compared to other treatments.

The TFC in dried peel was significantly affected by different drying methods (*p* < 0.05). As shown in Table 1, the peel treated with FD had the highest TFC value, followed by OD50, OD70, SD, and MD in sequence. The flavonoids could be degraded by thermal treatment and by some endogenous enzymes such as PPO and peroxidase (POD) [19]. The TFC in OD50 was slightly lower than that in FD (no significant difference at *p* > 0.05), suggesting that the heat temperature at 50 °C could be capable of deactivating the enzymes associated with the degradation of flavonoids, but not be high enough to significantly destroy flavonoids by thermal decomposition during drying processing. When the temperature is raised to 70 °C, some heat-sensitive flavonoids might be decomposed, thus resulting in lower TFC in OD70 relative to OD50 in the present study. The SD treatment was performed at ambient temperature which might be an appropriate temperature for the catalytic activity of some related enzymes such as PPO or POD, implying the flavonoids might be exposed to these high-activity enzymes for a long time, therefore, the TFC in SD treated peel was significantly lower than that in OD treated ones. It has been reported that the optimum temperature for the enzymatic activity of PPO and POD in *Rumex obtusifolius* L. is 30 and 25 °C, respectively [20], which is supportive of this assumption. In agreement with the present result, the TFC of physiologically dropped immature citrus fruit dried by hot air at 60 °C was not significantly different from that obtained from freeze drying, and both of them were higher than that treated with sun drying at a temperature around 25 °C [3]. Interestingly, the present result indicated that both the TPC and TFC values in the peel treated with MD were the smallest among all these five treatments. The microwave radiation with high energy can rapidly diffuse into the internal cells and be quickly absorbed by some molecules such as phenolics and flavonoids [21], thus causing their decomposition by breaking down covalent bonds, which might account for this observation. Similarly, Liu et al. reported that the microwave treatment contributed to the greatest losses of phenolics and antioxidant capacities in buckwheat samples relative to all other thermal treatments [22], and the microwave drying caused the greatest decrease in TPC of *Phyllanthus amarus* as compared to the sun and hot-air drying methods [23].

### 3.2. Effect of Different Drying Methods on the Contents of Individual Flavonoids

**Figure 1:**
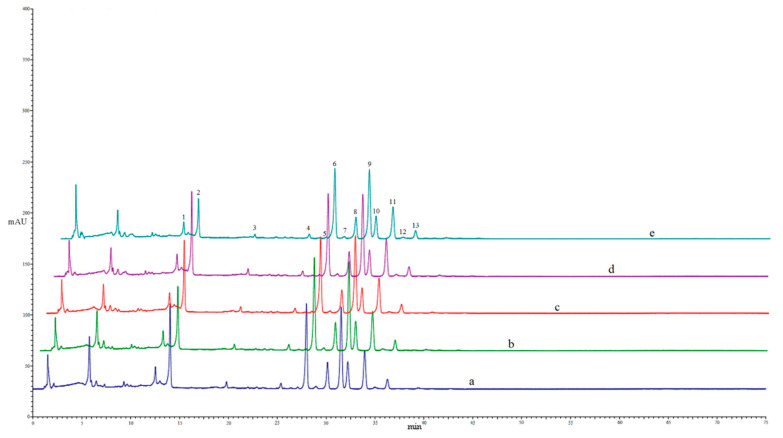
HPLC profiles of extracts from the navel orange peels dried by different drying methods. The letters from a to e, represent FD, SD, OD50, OD70, and MD, respectively. The number above the peak represent the following compounds: (1) narirutin, (2) hesperidin, (3) didymin, (4) isosinensetin, (5) 3,3′,4′,5,7,8-hexamethoxyflavone, (6) sinensetin, (7) 4′,5,7,8-tetramethoxyflavone, (8) 3,3′,4′,5,6,7-hexamethoxyflavone, (9) nobiletin, (10) 4′,5,6,7-tetramethoxyflavone, (11) 3,3′,4′,5,6,7,8-heptamethoxyflavone, (12) 5-hydroxy-6,7,3′,4′-tetramethoxyflavone, (13) tangeretin.

As shown in Figure 1, thirteen flavonoids involving three flavanone glycosides (FGs) and ten polymethoxyflavones (PMFs) were identified from the peel extract. The contents of these flavonoids were quantified by HPLC as shown in Table 2. The present study indicated that the most abundant FG in navel orange peel was hesperidin, followed by narirutin and didymin. The PMFs mainly consisted of sinensetin, nobiletin, 3,3′,4′,5,6,7,8-heptamethoxyflavone, 3,3′,4′,5,6,7-hexamethoxyflavone, and 4′,5,6,7-tetramethoxyflavone with a descending order in content, as revealed in Table 2. In line with the present result, previous phytochemical investigations have shown that hesperidin and narirutin are the two dominant flavanone glycosides present in the orange peel [6], and PMFs involving nobiletin, tangeretin, and sinesetin are abundant flavones in citrus peel [5]. The contents of these thirteen individual flavonoids from peels dried with different methods were compared in the present study. As shown in Table 2, the contents of PMFs were slightly influenced by different drying methods, which showed no significant statistical difference among all these different treatments. However, the MD treatment significantly reduced the content of all three FGs in peel extract compared with other treatments. The content of narirutin, hesperidin, and didymin was 6.22, 17.28, and 0.88 μg/mg DW, respectively, in the peel treated with MD, whereas it ranged from 8.17 to 9.57, 31.29 to 36.27, and 1.71 to 1.85, respectively, for other treatments.

Despite there being no significant difference in the content of FGs in peels treated with FD, SD, OD50, and OD70, the peel treated with SD contained the lowest amounts of both narirutin and hesperidin among them. This result was consistent with the TFC result showing that the peel treated with MD and SD contained the lowest and the second-lowest TFC, respectively. Previous studies indicated that microwave irradiation can cleave the ester and glycosidic bond of phenolics in citrus peel [24], and have the ability to hydrolyze rice starch by breaking down C-O-C covalent linkage between monosaccharides [25]. Therefore, we assumed that the glycosidic bond of FGs might be vulnerable to the MD treatment, which might initially break down upon absorbing the microwave irradiation, then inducing the further degradation of FGs, thus making the peel treated with MD contain low amounts of FGs as shown in Table 2. Moreover, the PMFs’ structural differences from FGs mainly by the absence of sugar moiety were slightly influenced by MD treatment, which further supported this assumption. However, more studies should be carried out to elucidate the degradation mechanisms of FGs. The present result also revealed that the PMFs were much more stable under all these drying conditions, which was in line with a previous study showing that the methylation treatments improved the stability of flavonoids [26].

### 3.3. Evaluation of Antioxidant Capacity

The antioxidant capacity of peels dried with different methods was evaluated by DPPH, ABTS, and FRAP assays. As shown in Table 3, the extract from peel treated with OD50 demonstrated the most potent capacity in scavenging both DPPH and ABTS radicals, and the extract from peel treated with OD70 was superior in FRAP assay when compared to other treatments. In contrast, the extract from the peel treated with FD had the highest IC_50_ value in both DPPH and ABTS assays, and the SD treatment made the peel extract less powerful in reducing ferric (III) to ferrous (II) ions as revealed in the FRAP assay relative to other treatments. Both DPPH and ABTS are stable free radicals, the scavenging of these two radicals is mainly based on the electron transfer and the hydrogen atom transfer reaction mechanisms [27]. Being different from the radical-scavenging assays, the FRAP assay is commonly used to evaluate the overall reducing ability of antioxidants by measuring the reduction of ferric (III) to ferrous (II) ions [28]. The antioxidant capacity evaluated by the DPPH assay is much more consistent with that by the ABTS assay but different from that obtained by the FRAP assay in the present study, which might be due to the discrepancy in the mechanisms of different antioxidant assays. The present result also supported that more than one antioxidant assay should be performed to comprehensively evaluate the antioxidant capacity because a single antioxidant assay is not sufficient to measure the various modes of action of antioxidants [29]. Similar to the present study, the extract from lemon pomace dried by hot air exhibited higher antioxidant capacity in scavenging DPPH radical than that dried by freeze drying [8]. However, Sun et al. reported that the antioxidant capacity of physiologically dropped immature citrus fruits dried by freeze drying was higher when compared to those dried by hot air or sun drying in both DPPH and FRAP assays [3], which was inconsistent with the present study. These differences could be attributed to the different drying conditions and the extraction methods applied, as well as the different citrus cultivars used.

### 3.4. Pearson’s Correlation Analysis

**Figure 2:**
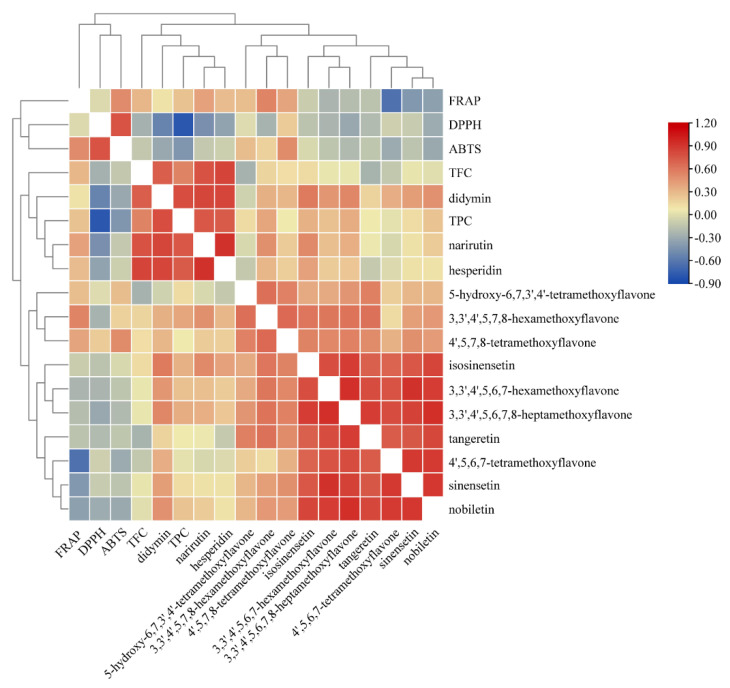
Pearson’s correlation analysis among all variables under investigation.

Pearson’s correlation analysis was used to evaluate the relationships among all variables in the present study. The result of Pearson’s correlation analysis is usually expressed as a correlation coefficient value, which is represented as *r*. The values of *r* range between −1.000 and 1.000. A correlation of −1.000 shows a perfect negative correlation, while a correlation of 1.000 shows a perfect positive correlation [30]. As shown in Figure 2, TFC was highly correlated with all three FGs involving narirutin (*r* = 0.777), hesperidin (*r* = 0.843) and didymin (*r* = 0.795) at *p* < 0.01, suggesting that these three FGs should be the major flavonoids present in peel extract. In addition, the consistent variation trend of DPPH and ABTS in response to different drying methods was found in the present study, which was supported by the strong positive correlation between DPPH and ABTS (*r* = 0.770, *p* < 0.01). Similarly, several previous studies reported a high positive correlation between them [13]. Moreover, DPPH was highly negatively correlated with TPC (*r* = −0.793, *p* < 0.01), and moderately negatively correlated with TFC (*r* = −0.455, *p* < 0.01), suggesting that the phenolics rather than flavonoids should be the main contributors in scavenging the DPPH radical in peel extract. DPPH was expressed as IC_50_ in the present study, the lower value of it representing the higher antioxidant capacity of extract, therefore, the negative correlation coefficient value was obtained. FRAP showed a weak correlation with both TPC (*r* = 0.258, *p* < 0.01) and TFC (*r* = 0.313, *p* < 0.01), indicating that the antioxidant capacity evaluated by FRAP assay could be ascribed to the synergistic effects among antioxidants, or come from other compounds such as polysaccharides, limonoids, and ascorbic acid. Consistent with the present study, a very weak correlation (*r* = 0.118) was observed between FRAP and TPC of extracts from the pomelo peel [14].

Actually, the antioxidant activity might not always correlate with phenolic content, and the presence of other bioactive compounds such as terpenoids and limonoids in citrus peel could act as an antioxidant [5]. The high correlations between DPPH with didymin (*r* = −0.529, *p* < 0.01) and between FRAP with 3,3′,4′,5,6,7-hexamethoxyflavone were found in the present study, suggesting these two compounds might be potent antioxidants in the orange peel. In line with our results, a high correlation between didymin and DPPH (*r* = 0.83, *p* < 0.01) was found in a recent report studying immature dropped *Citrus sinensis* L. Osbeck fruits [31].

## 4. Conclusions

This study comprehensively studied the effects of five different drying methods, namely FD, SD, OD50, OD70, and MD, on the chemical compositions and antioxidant capacity of navel orange peel. The peel treated with OD70 had the highest TPC and FRAP values, and the peel treated with OD50 exhibited the best antioxidant capacity in both DPPH and ABTS assays, when compared to other treatments. The highest TFC was obtained in peel dried by the FD method, which was slightly higher than that in the peel treated with OD50, but there was no significant difference between them (*p* > 0.05). The peel dried by the MD method had the highest extracting yield but contained the lowest TPC and TFC, as well as the lowest contents of three FGs involving narirutin, hesperidin, and didymin, relative to other drying methods. HPLC analysis identified thirteen flavonoids (three FGs and ten PMFs) in the peel extract, and all PMFs were stable during the drying process, showing slight variation in all five different drying methods applied. Pearson’s correlation analysis was further employed to test the relationships among all variables. The results revealed here allow us to recommend the use of OD50 or OD70 for the drying of orange peel, both of which help the maintenance of bioactive compounds in the peel and improve its antioxidant capacity. This recommendation is supported as well since the OD drying method is a relatively inexpensive and user-friendly method.

## Figures and Tables

**Table 1:** Extracting yield, TPC and TFC of extracts from navel orange peel dried by different methods.

Drying Methods	Yield (%)	TPC (μM GAE/g DW)	TFC (μM QE/g DW)
FD	34.59 ± 0.27 ^b^	75.42 ± 0.41 ^c^	183.06 ± 1.57 ^a^
SD	32.44 ± 0.31 ^d^	77.75 ± 0.62 ^b^	160.41 ± 1.22 ^c^
OD50	33.33 ± 0.15 ^c^	78.93 ± 1.04 ^b^	182.57 ± 3.21 ^a^
OD70	32.08 ± 0.11 ^d^	81.36 ± 0.91 ^a^	168.14 ± 0.92 ^b^
MD	35.63 ± 0.12 ^a^	71.72 ± 0.73 ^d^	139.93 ± 3.28 ^d^

The data are reported as average ± SD (three replicates). The different superscript lowercase letters within the same column indicate significant statistical difference (*p* < 0.05). GAE: Gallic acid equivalent. QE: Quercetin equivalent. DW: Dry weight. TPC: Total phenolic content. TFC: Total flavonoid content. FD: Freeze drying. SD: Shade drying. OD50: Hot-air oven drying at 50 °C. OD70: Hot-air oven drying at 70 °C. MD: Microwave drying.

**Table 2:** HPLC quantification analysis of flavonoids (μg/mg DW) in extracts from the orange peels dried by different methods.

NO	Flavonoids	FD	SD	OD50	OD70	MD
1	narirutin	9.10 ± 0.32 ^a^	8.17 ± 0.80 ^a^	9.13 ± 0.70 ^a^	9.57 ± 0.32 ^a^	6.22 ± 1.11 ^b^
2	hesperidin	36.27 ± 3.25 ^a^	31.29 ± 1.19 ^a^	32.79 ± 2.14 ^a^	35.51 ± 1.22 ^a^	17.28 ± 2.91 ^b^
3	didymin	1.71 ± 0.05 ^a^	1.72 ± 0.26 ^a^	1.85 ± 0.35 ^a^	1.82 ± 0.14 ^a^	0.88 ± 0.25 ^b^
4	isosinensetin	0.12 ± 0.01 ^a^	0.12 ± 0.01 ^a^	0.11 ± 0.02 ^a^	0.12 ± 0.01 ^a^	0.10 ± 0.01 ^a^
5	3,3’,4’,5,7,8-hexamethoxyflavone	0.05 ± 0.01 ^a^	0.04 ± 0.01 ^a^	0.05 ± 0.01 ^a^	0.06 ± 0.01 ^a^	0.05 ± 0.01 ^a^
6	sinensetin	1.59 ± 0.04 ^a^	1.62 ± 0.01 ^a^	1.59 ± 0.09 ^a^	1.56 ± 0.05 ^a^	1.56 ± 0.07 ^a^
7	4’,5,7,8-tetramethoxyflavone	0.05 ± 0.01 ^a^	0.04 ± 0.01 ^a^	0.05 ± 0.01 ^a^	0.05 ± 0.01 ^a^	0.05 ± 0.01 ^a^
8	3,3’,4’,5,6,7-hexamethoxyflavone	0.58 ± 0.01 ^a^	0.59 ± 0.01 ^a^	0.59 ± 0.03 ^a^	0.59 ± 0.02 ^a^	0.58 ± 0.02 ^a^
9	nobiletin	1.09 ± 0.03 ^a^	1.13 ± 0.04 ^a^	1.12 ± 0.06 ^a^	1.10 ± 0.04 ^a^	1.09 ± 0.05 ^a^
10	4’,5,6,7-tetramethoxyflavone	0.52 ± 0.01 ^a^	0.56 ± 0.02 ^a^	0.53 ± 0.03 ^a^	0.51 ± 0.02 ^a^	0.52 ± 0.04 ^a^
11	3,3’,4’,5,6,7,8-heptamethoxyflavone	0.90 ± 0.01 ^a^	0.93 ± 0.02 ^a^	0.92 ± 0.05 ^a^	0.92 ± 0.03 ^a^	0.89 ± 0.04 ^a^
12	5-hydroxy-6,7,3’,4’-tetramethoxyflavone	0.09 ± 0.01 ^a^	0.08 ± 0.01 ^a^	0.08 ± 0.01 ^a^	0.11 ± 0.03 ^a^	0.10 ± 0.01 ^a^
13	tangeretin	0.13 ± 0.01 ^a^	0.14 ± 0.01 ^a^	0.15 ± 0.01 ^a^	0.14 ± 0.02 ^a^	0.14 ± 0.01 ^a^

The data are reported as averages ± SD (three replicates). Different superscript lowercase letters within the same line indicate significant statistical difference (*p* < 0.05). FD: Freeze drying. SD: Shade drying. OD50: Hot-air oven drying at 50 °C. OD70: Hot-air oven drying at 70 °C. MD: Microwave drying.

**Table 3:** Antioxidant capacity of extracts from the navel orange peels dried by different methods via DPPH, ABTS, and FRAP assays.

Drying Methods	DPPH (IC_50_ mg/mL)	ABTS (IC_50_ mg/mL)	FRAP (μg VC/mg)
FD	1.37 ± 0.01 ^a^	0.32 ± 0.01 ^a^	4.03 ± 0.04 ^b^
SD	1.23 ± 0.02 ^b^	0.24 ± 0.01 ^c^	3.39 ± 0.05 ^d^
OD50	1.15 ± 0.04 ^c^	0.23 ± 0.00 ^c^	3.92 ± 0.03 ^bc^
OD70	1.18 ± 0.01 ^bc^	0.28 ± 0.01 ^b^	4.27 ± 0.09 ^a^
MD	1.34 ± 0.01 ^a^	0.29 ± 0.01 ^b^	3.86 ± 0.06 ^c^
VC (positive control)	0.0033 ± 0.0003 ^d^	0.0029 ± 0.0001 ^d^	—

The data are reported as average ± SD (three replicates). The different superscript lowercase letters within the same column indicate significant statistical difference (*p* < 0.05). VC: Vitamin C. —: Not applicable. FD: Freeze drying. SD: Shade drying. OD50: Hot-air oven drying at 50 °C. OD70: Hot-air oven drying at 70 °C. MD: Microwave drying. DPPH: 2,2-Diphenyl-1-picrylhydrazyl free radical. ABTS: 2,2′-Azinobis-(3-ethylbenzthiazoline-6-sulphonate) free radical. FRAP: Ferric reducing antioxidant power.

## Data Availability

Data are contained within the article and supplementary material.

## Supplementary Materials

The following supporting information can be downloaded at: https://www.mdpi.com/article/10.3390/antiox11081543/s1. Table S1. The retention time, regression parameters, and linear range of standard compounds analyzed by HPLC. Figures S1–S13. HPLC profile (340 nm) of flavonids (**1**-**13**) obtained from the navel orange peel.

## References

[1] Zhang J., Zhang J.Y., Shan Y.X., Guo C., He L., Zhang L.Y., Ling W., Liang Y., Zhong B.L. (2022). Effect of harvest time on the chemical composition and antioxidant capacity of Gannan navel orange (*Citrus sinensis* L. Osbeck ‘Newhall’) juice. J. Integr. Agr..

[2] Guo C., Shan Y., Yang Z., Zhang L., Ling W., Liang Y., Ouyang Z., Zhong B., Zhang J. (2020). Chemical composition, antioxidant, antibacterial, and tyrosinase inhibition activity of extracts from Newhall navel orange (*Citrus sinensis* Osbeck cv. Newhall) Peel. J. Sci. Food Agric..

[3] Sun Y., Shen Y., Liu D., Ye X. (2015). Effects of drying methods on phytochemical compounds and antioxidant activity of physiologically dropped un-Matured citrus fruits. LWT-Food Sci. Technol..

[4] Ghanem Romdhane N., Bonazzi C., Kechaou N., Mihoubi N.B. (2015). Effect of air-drying temperature on kinetics of quality attributes of lemon (*Citrus limon* cv. Lunari) peels. Dry. Technol..

[5] Saini R.K., Ranjit A., Sharma K., Prasad P., Shang X., Gowda K.G.M., Keum Y.S. (2022). Bioactive compounds of citrus fruits: A review of composition and health benefits of carotenoids, flavonoids, limonoids, and terpenes. Antioxidants.

[6] Pereira R.M., López B.G.C., Diniz S.N., Antunes A.A., Garcia D.M., Oliveira C.R., Marcucci M.C. (2017). Quantification of flavonoids in Brazilian orange peels and industrial orange juice processing wastes. Agric. Sci..

[7] Deng L.Z., Mujumdar A.S., Yang W.X., Zhang Q., Zheng Z.A., Wu M., Xiao H.W. (2020). Hot air impingement drying kinetics and quality attributes of orange peel. J. Food Process. Pres..

[8] Papoutsis K., Pristijono P., Golding J.B., Stathopoulos C.E., Bowyer M.C., Scarlett C.J., Vuong Q.V. (2017). Effect of vacuum-drying, hot air-drying and freeze-drying on polyphenols and antioxidant capacity of lemon (*Citrus limon*) pomace aqueous extracts. Int. J. Food Sci. Technol..

[9] Garau M.C., Simal S., Rossello C., Femenia A. (2007). Effect of air-drying temperature on physico-chemical properties of dietary fibre and antioxidant capacity of orange (*Citrus aurantium* v. Canoneta) by-products. Food Chem..

[10] Kammoun Bejar A., Kechaou N., Boudhrioua Mihoubi N. (2011). Effect of microwave treatment on physical and functional properties of orange (*Citrus Sinensis*) peel and leaves. J. Food Process. Technol..

[11] Ledesma-Escobar C.A., Priego-Capote F., Luque de Castro M.D. (2016). Comparative study of the effect of sample pretreatment and extraction on the determination of flavonoids from lemon (*Citrus limon*). PLoS ONE.

[12] Zhang J., Zhang J., Kaliaperumal K., Zhong B. (2022). Variations of the chemical composition of *Citrus sinensis* Osbeck cv. Newhall fruit in relation to the symptom severity of Huanglongbing. J. Food Compos. Anal..

[13] Dudonne S., Vitrac X., Coutiere P., Woillez M., Mérillon J.M. (2009). Comparative study of antioxidant properties and total phenolic content of 30 plant extracts of industrial interest using DPPH, ABTS, FRAP, SOD, and ORAC assays. J. Agric. Food Chem..

[14] Abd Rahman N.F., Shamsudin R., Ismail A., Shah N.N.A.K., Varith J. (2018). Effects of drying methods on total phenolic contents and antioxidant capacity of the pomelo (*Citrus grandis* (L.) Osbeck) peels. Innov. Food Sci. Emerg..

[15] Jia Y., Khalifa I., Hu L., Zhu W., Li J., Li K., Li C. (2019). Influence of three different drying techniques on persimmon chips’ characteristics: A comparison study among hot-air, combined hot-air-microwave, and vacuum-freeze drying techniques. Food Bioprod. Process..

[16] Shu B., Wu G., Wang Z., Wang J., Huang F., Dong L., Zhang R., Wang Y., Su D. (2020). the effect of microwave vacuum drying process on citrus: Drying kinetics, physicochemical composition and antioxidant activity of dried citrus (*Citrus reticulata* Blanco) peel. J. Food Meas. Charact..

[17] Tian Y., Zhao Y., Huang J., Zeng H., Zheng B. (2016). Effects of different drying methods on the product quality and volatile compounds of whole Shiitake mushrooms. Food Chem..

[18] Krapfenbauer G., Kinner M., Gössinger M., Schönlechner R., Berghofer E. (2006). Effect of thermal treatment on the quality of cloudy apple juice. J. Agric. Food Chem..

[19] Almeida J.R., D’Amico E., Preuss A., Carbone F., de Vos C.R., Deiml B., Mourgues F., Perrotta G., Fischer T.C., Bovy A.G. (2007). Characterization of major enzymes and genes involved in flavonoid and proanthocyanidin biosynthesis during fruit development in strawberry (*Fragaria* × *ananassa*). Arch. Biochem. Biophs..

[20] Alici E.H., Arabaci G. (2016). Determination of SOD, POD, PPO and cat enzyme activities in *Rumex obtusifolius* L.. Annu. Res. Rev. Biol..

[21] Flórez N., Conde E., Domínguez H. (2015). Microwave assisted water extraction of plant compounds. J. Chem. Technol. Biot..

[22] Liu Y., Cai C., Yao Y., Xu B. (2019). Alteration of phenolic profiles and antioxidant capacities of common buckwheat and tartary buckwheat produced in China upon thermal processing. J. Sci. Food Agric..

[23] Lim Y.Y., Murtijaya J. (2007). Antioxidant properties of *Phyllanthus amarus* extracts as affected by different drying methods. LWT-Food Sci. Technol..

[24] Hayat K., Zhang X., Chen H., Xia S., Jia C., Zhong F. (2010). Liberation and separation of phenolic compounds from citrus mandarin peels by microwave heating and its effect on antioxidant activity. Sep. Purif. Technol..

[25] Iris K.M., Fan J., Budarin V.L., Bouxin F.P., Clark J.H., Tsang D.C. (2020). NaCl-promoted phase transition and glycosidic bond cleavage under microwave heating for energy-efficient biorefinery of rice starch. Green Chem..

[26] Walle T. (2009). Methylation of dietary flavones increases their metabolic stability and chemopreventive effects. Int. J Mol. Sci..

[27] Schaich K.M., Tian X., Xie J. (2015). Hurdles and pitfalls in measuring antioxidant efficacy: A critical evaluation of ABTS, DPPH, and ORAC assays. J. Funct. Foods.

[28] Li H.B., Wong C.C., Cheng K.W., Chen F. (2008). Antioxidant properties in vitro and total phenolic contents in methanol extracts from medicinal plants. LWT-Food Sci. Technol..

[29] Rakholiya K., Kaneria M., Chanda S. (2011). Vegetable and fruit peels as a novel source of antioxidants. J. Med. Plants Res..

[30] Mukaka M.M. (2012). A guide to appropriate use of correlation coefficient in medical research. Malawi Med. J..

[31] Kumar D., Ladaniya M.S., Gurjar M., Kumar S. (2022). Impact of drying methods on natural antioxidants, phenols and flavanones of immature dropped *Citrus sinensis* L. Osbeck fruits. Sci. Rep..

