# Supplementary material for: Variations of Bioactive Phytochemicals and Antioxidant Capacity of Navel Orange Peel in Response to Different Drying Methods
Source: Antioxidants (Basel). 2022 Aug 9;11(8):1543. doi: 10.3390/antiox11081543 (PMC9404947; doi:10.3390/antiox11081543)
Supplement: Supplementary file 1 [file antioxidants-11-01543-s001.zip › antioxidants-1848097-supplementary.pdf]

Supplementary material

Variations of bioactive phytochemicals and antioxidant capacity of navel orange peel in response to different drying methods

Chunling Lai[1], Yan Liang[1,*], Linyan Zhang[1], Jiangjiang Huang[1], Kumaravel Kaliaperumal[1], Yueming Jiang[1,2], Jun Zhang[1,2,*]

[1] National Engineering Research Centre of Navel Orange, Gannan Normal University, Ganzhou 341000, China

[2] Key Laboratory of Plant Resources Conservation and Sustainable Utilization, South China Botanical Garden, Chinese Academy of Science, Guangzhou, 510650, China;

* Correspondence: bri71527152@outlook.com (J.Z.); zjhxy110@126.com (Y.L.)

Table S1. The retention time, regression parameters, and linear range of standard compounds analyzed by HPLC

Peaks	Flavonoids	Retention time (min)	Regression equation (340 nm)	Regression coefficient (R^2)	Linear range (μg/mL)	Resolution ($R_{a,b}$)
1	narirutin	12.231	y = 3179.2x - 3356.3	1	15.6-500	$R_{1,2}$ = 4.75
2	hesperidin	13.759	y = 2069.7x + 1425	1	7.8-125	$R_{2,3}$ = 17.45
3	didymin	19.445	y = 4216.1x + 31195	0.9999	32.2-1000	$R_{3,4}$ = 16.73
4	isosinensetin	25.353	y = 81172x+48149	0.9996	1.9-32.2	$R_{4,5}$ = 5.73
5	3,3′,4′,5,7,8-hexamethoxyflavone	27.052	y = 30903x + 15488	1	15.6-125	$R_{5,6}$ = 1.63
6	sinensetin	27.894	y = 55831x + 68257	1	15.6-125	$R_{6,7}$ = 1.72
7	4',5,7,8-tetramethoxyflavone	28.770	y = 47762x + 43908	1	15.6-125	$R_{7,8}$ = 3.41
8	3,3′,4′,5,6,7-hexamethoxyflavone	30.060	y = 54797x - 29076	0.9999	15.6-125	$R_{8,9}$ = 3.77
9	nobiletin	31.367	y = 86931x + 18267	0.9999	15.6-125	$R_{9,10}$ = 1.71
10	4',5,6,7-tetramethoxyflavone	32.041	y = 65269x + 70383	0.9999	15.6-125	$R_{10,11}$ = 3.69
11	3,3',4',5,6,7,8-heptamethoxyflavone	33.810	y = 52845x - 24248	1	15.6-500	$R_{11,12}$ = 2.30
12	5-hydroxy-6,7,3',4'-tetramethoxyflavone	34.509	y = 49367x - 14376	1	15.6-125	$R_{12,13}$ = 3.31
13	tangeretin	36.089	y = 85710x + 12989	1	15.6-62.5	

$R_{a,b}$ represents resolution between peaks a and b.

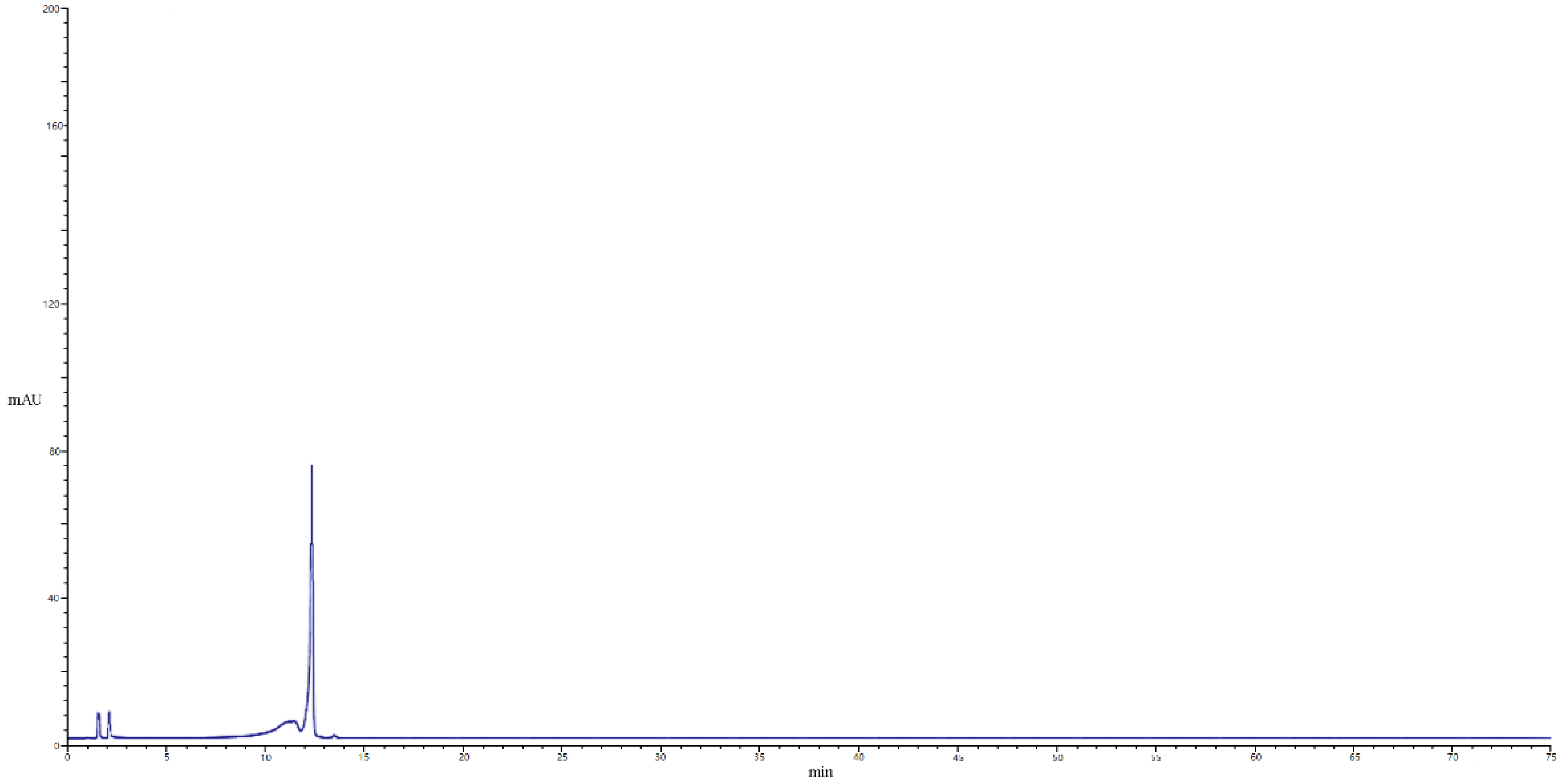

Figure S1: HPLC profile (340 nm) of narirutin (**1**) obtained from the navel orange peel.

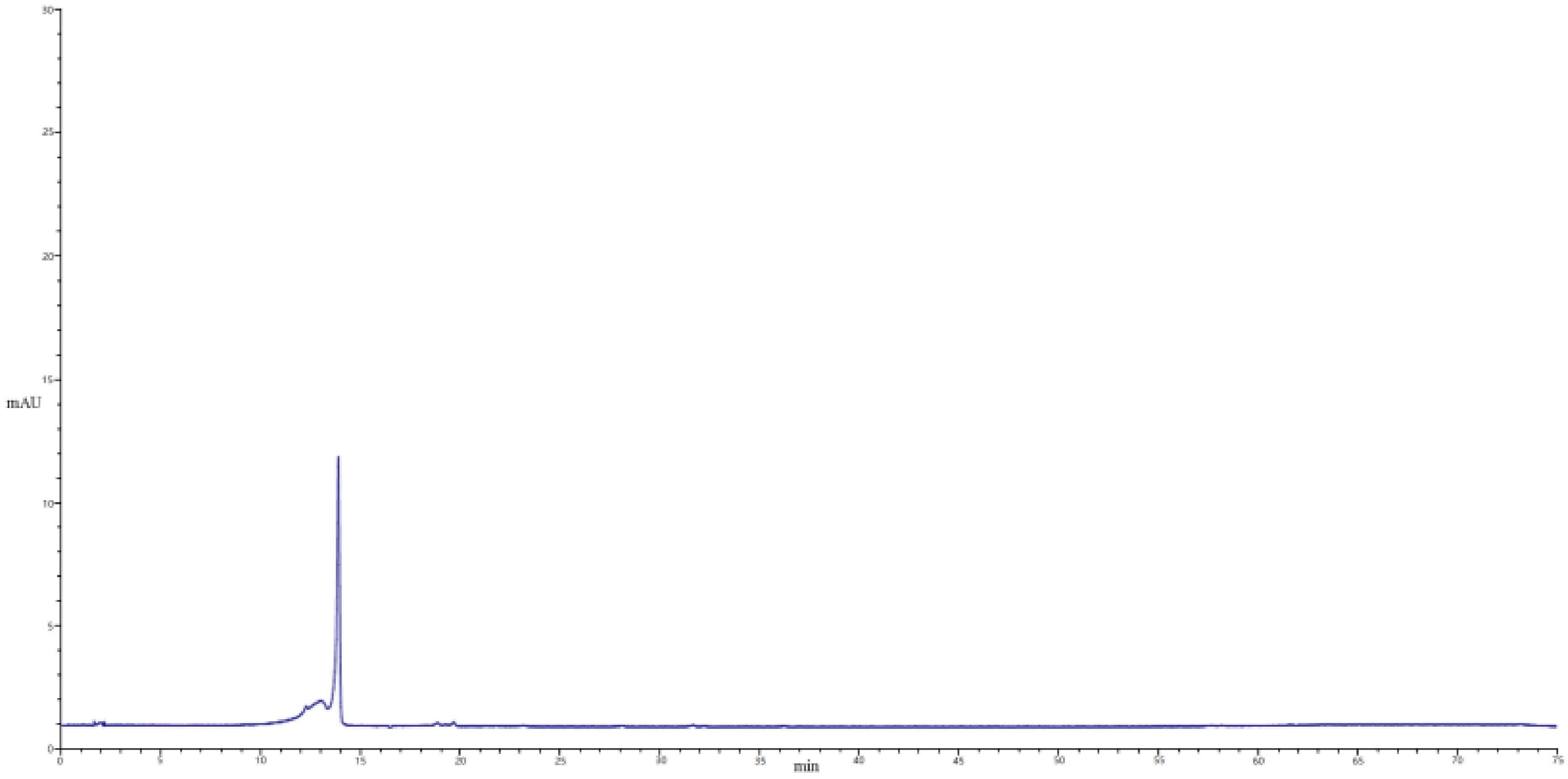

Figure S2: HPLC profile (340 nm) of hesperidin (**2**) obtained from the navel orange peel.

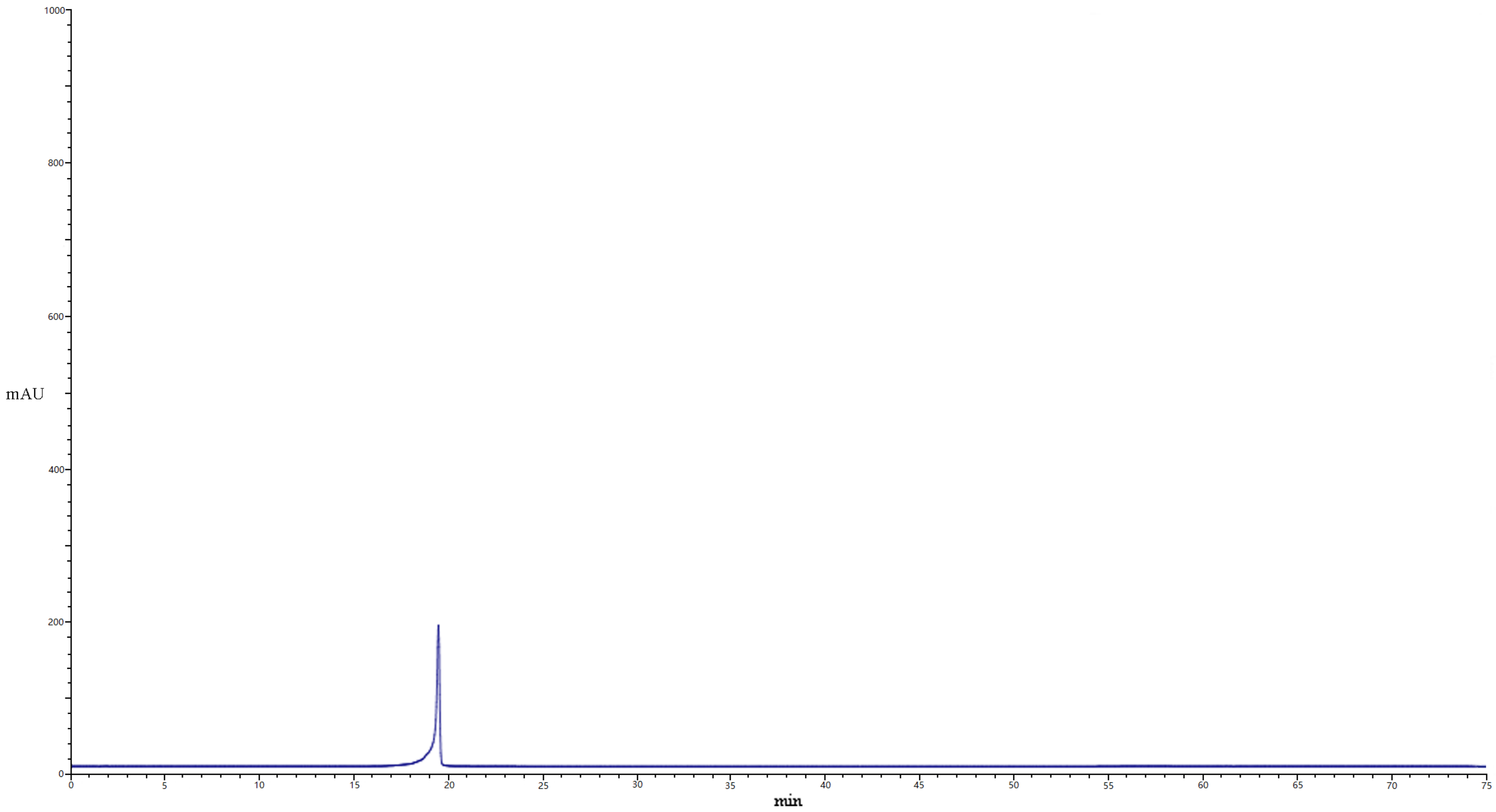

Figure S3: HPLC profile (340 nm) of didymin (**3**) obtained from the navel orange peel.

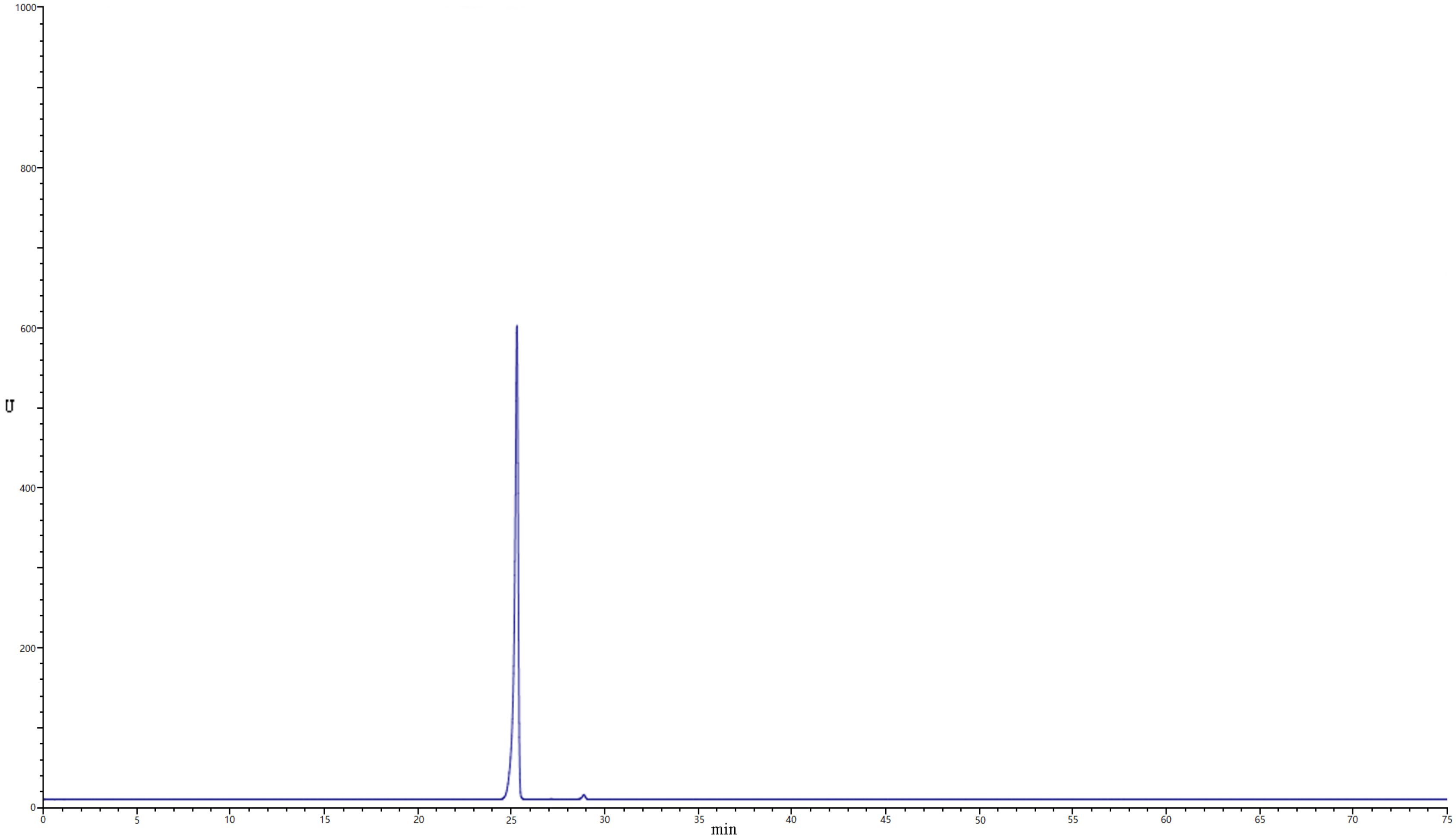

Figure S4: HPLC profile (340 nm) of isosinensetin (**4**) obtained from the navel orange peel.

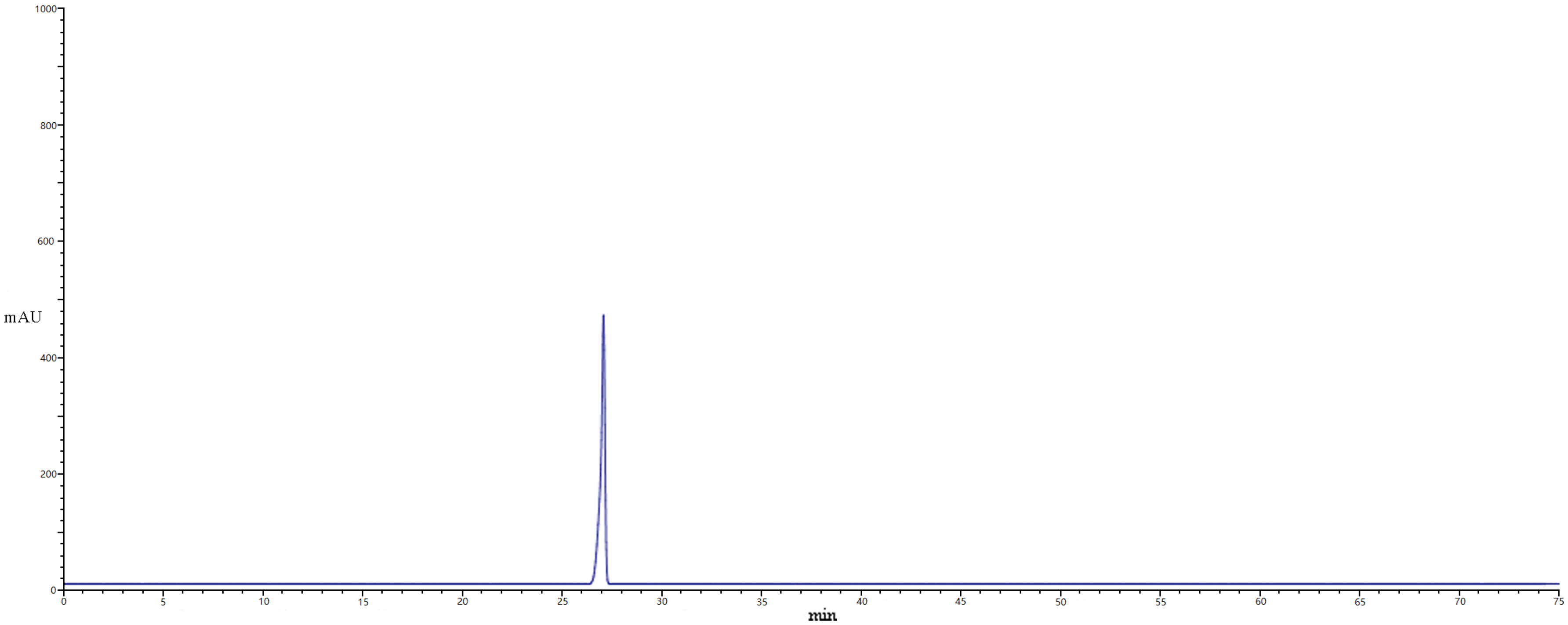

Figure S5: HPLC profile (340 nm) of 3,3',4',5,7,8-hexamethoxyflavone (**5**) obtained from the navel orange peel.

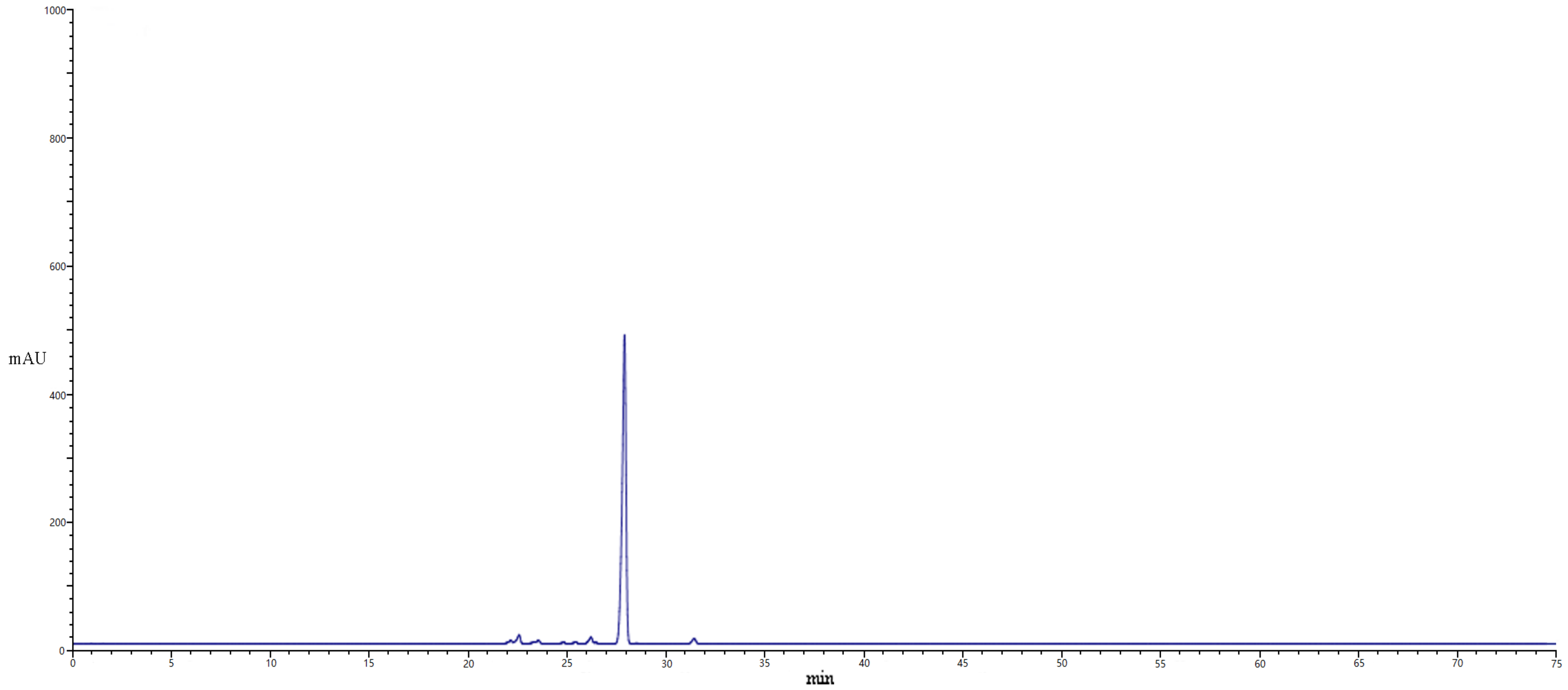

Figure S6: HPLC profile (340 nm) of sinensetin (**6**) obtained from the navel orange peel.

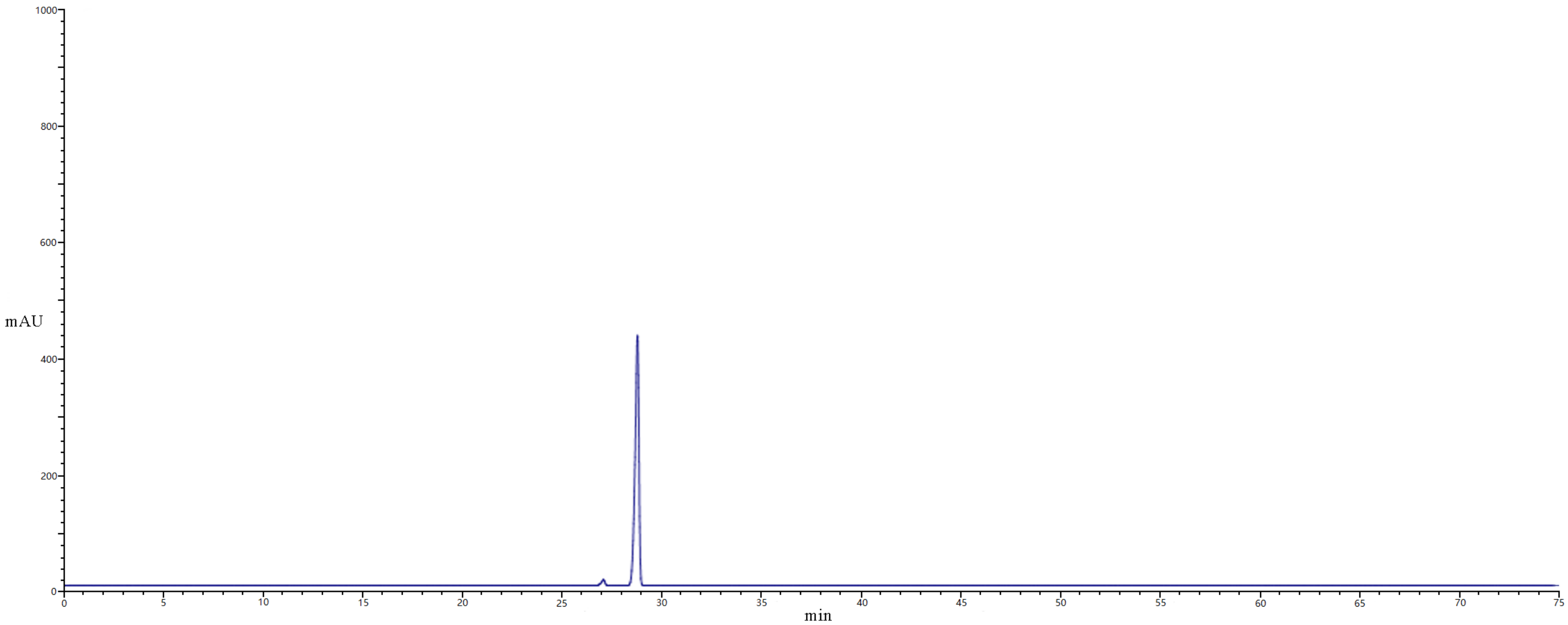

Figure S7: HPLC profile (340 nm) of 4',5,7,8-tetramethoxyflavone (**7**) obtained from the navel orange peel.

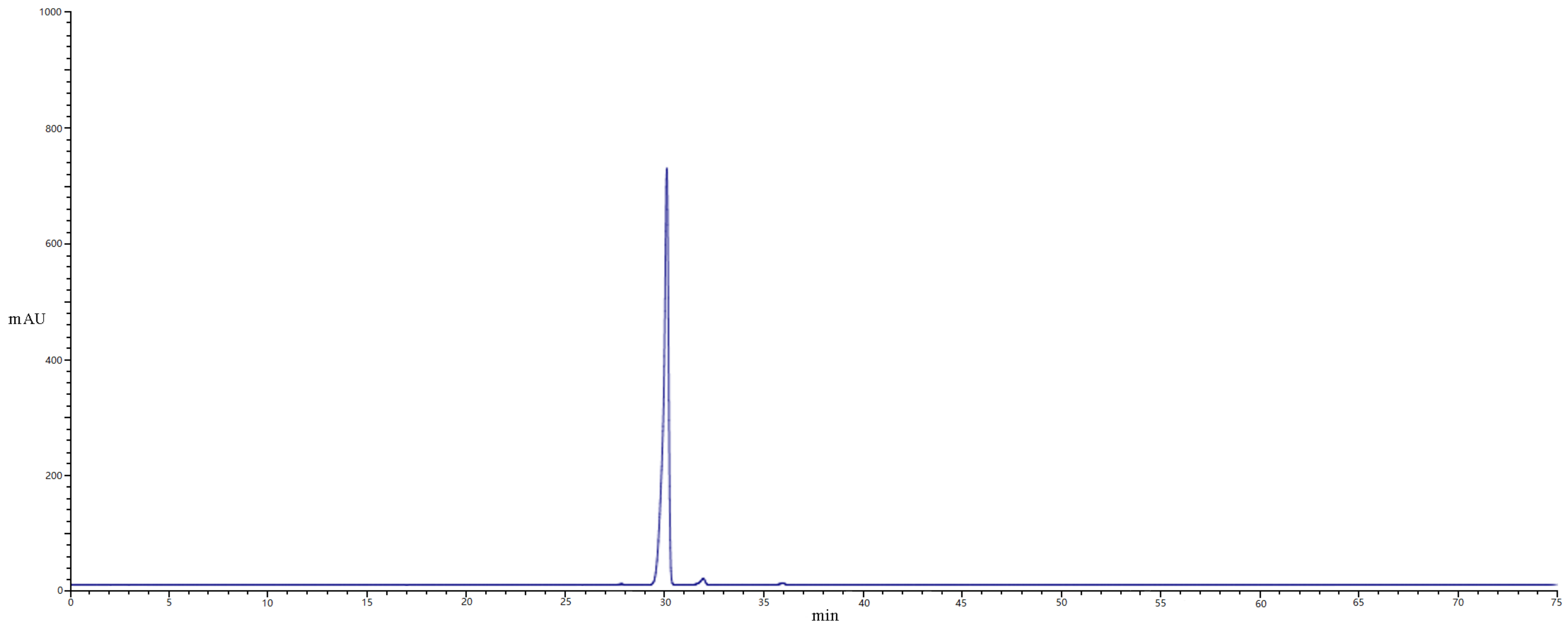

Figure S8: HPLC profile (340 nm) of 3,3',4',5,6,7-hexamethoxyflavone (**8**) obtained from the navel orange peel.

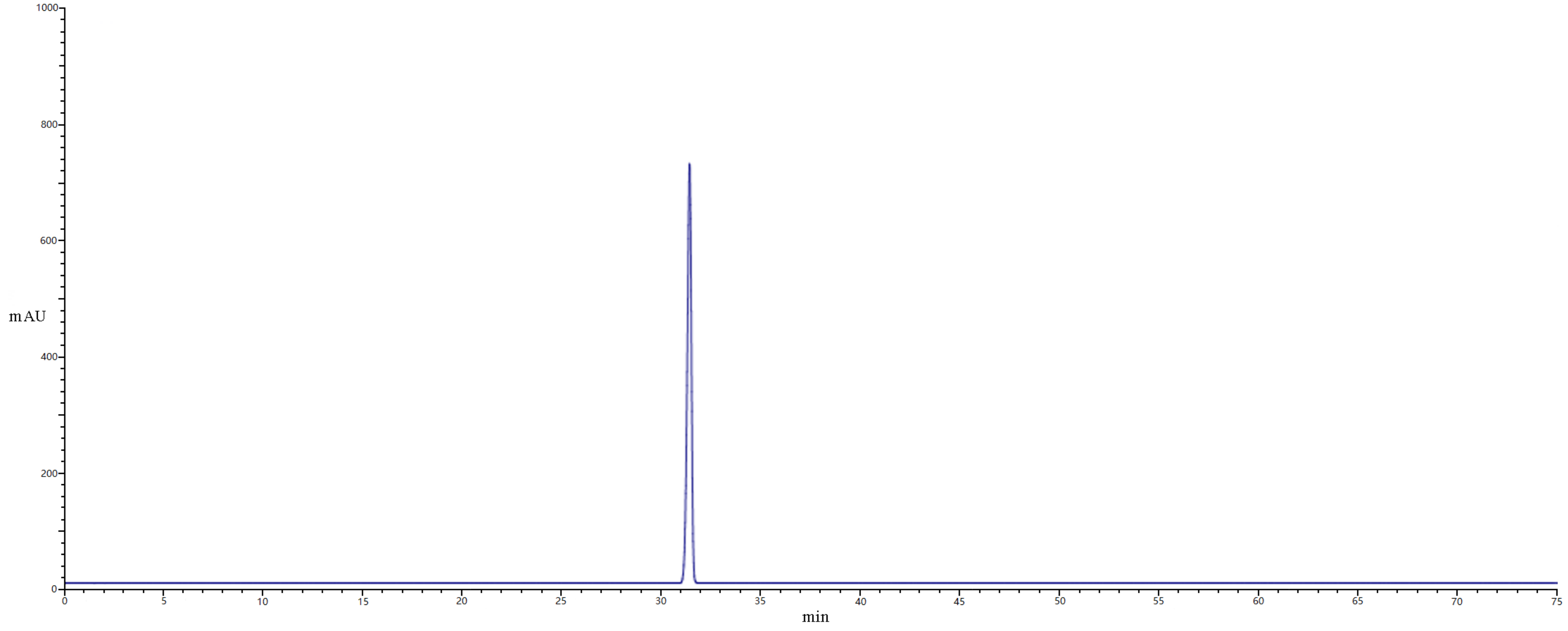

Figure S9: HPLC profile (340 nm) of nobiletin (**9**) obtained from the navel orange peel.

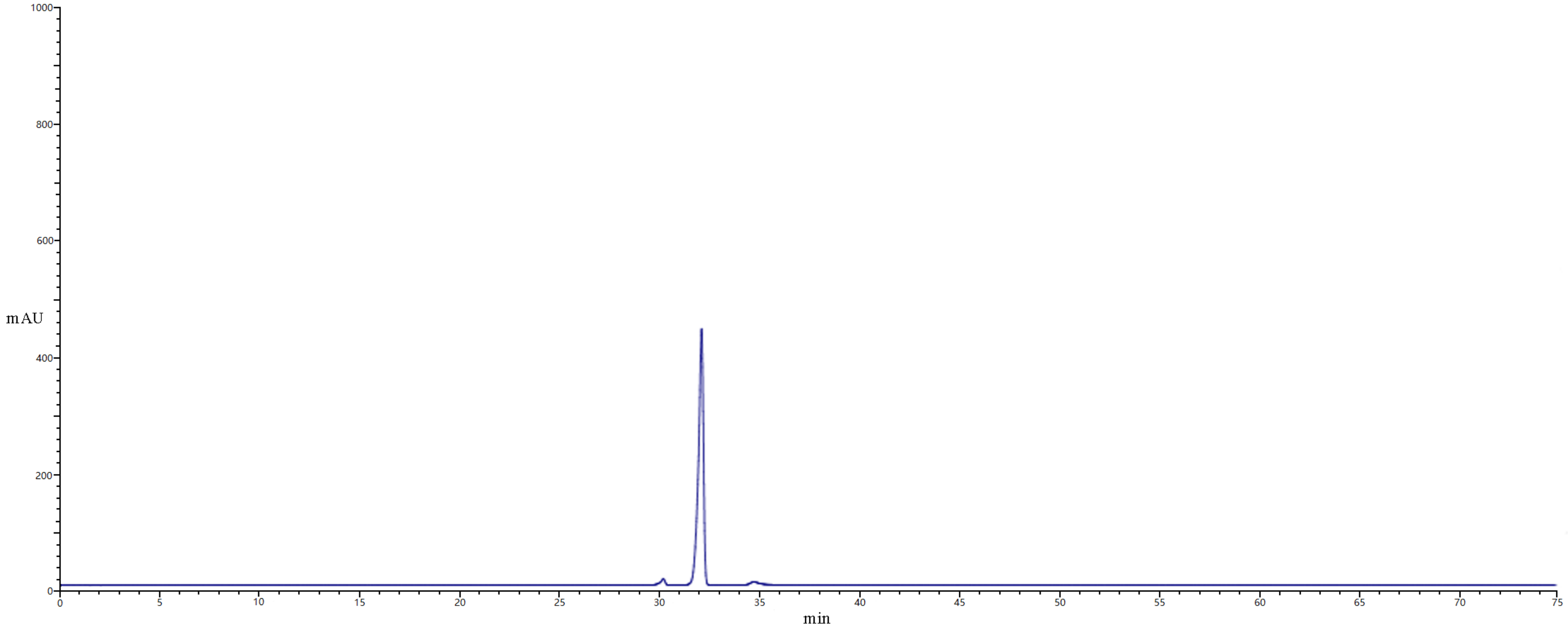

Figure S10: HPLC profile (340 nm) of 4',5,6,7-tetramethoxyflavone (**10**) obtained from the navel orange peel.

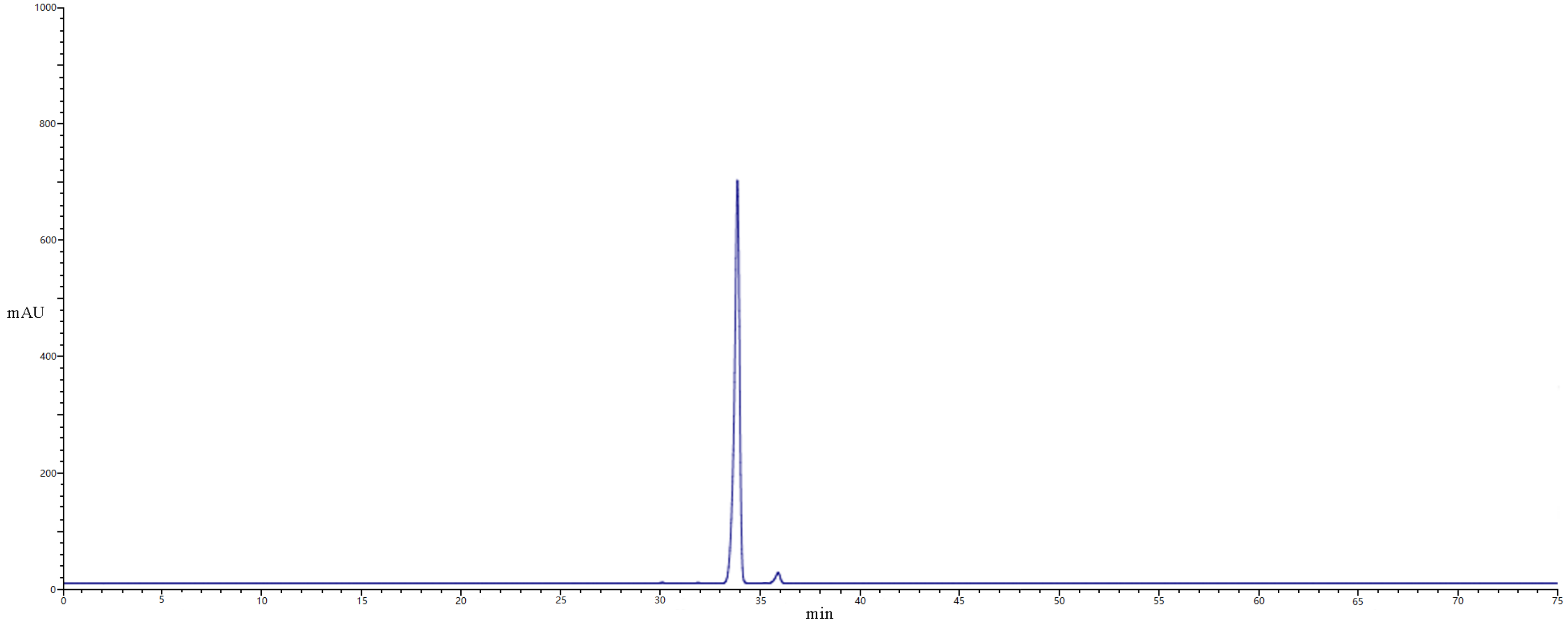

Figure S11: HPLC profile (340 nm) of 3,3',4',5,6,7,8-heptamethoxyflavone (**11**) obtained from the navel orange peel.

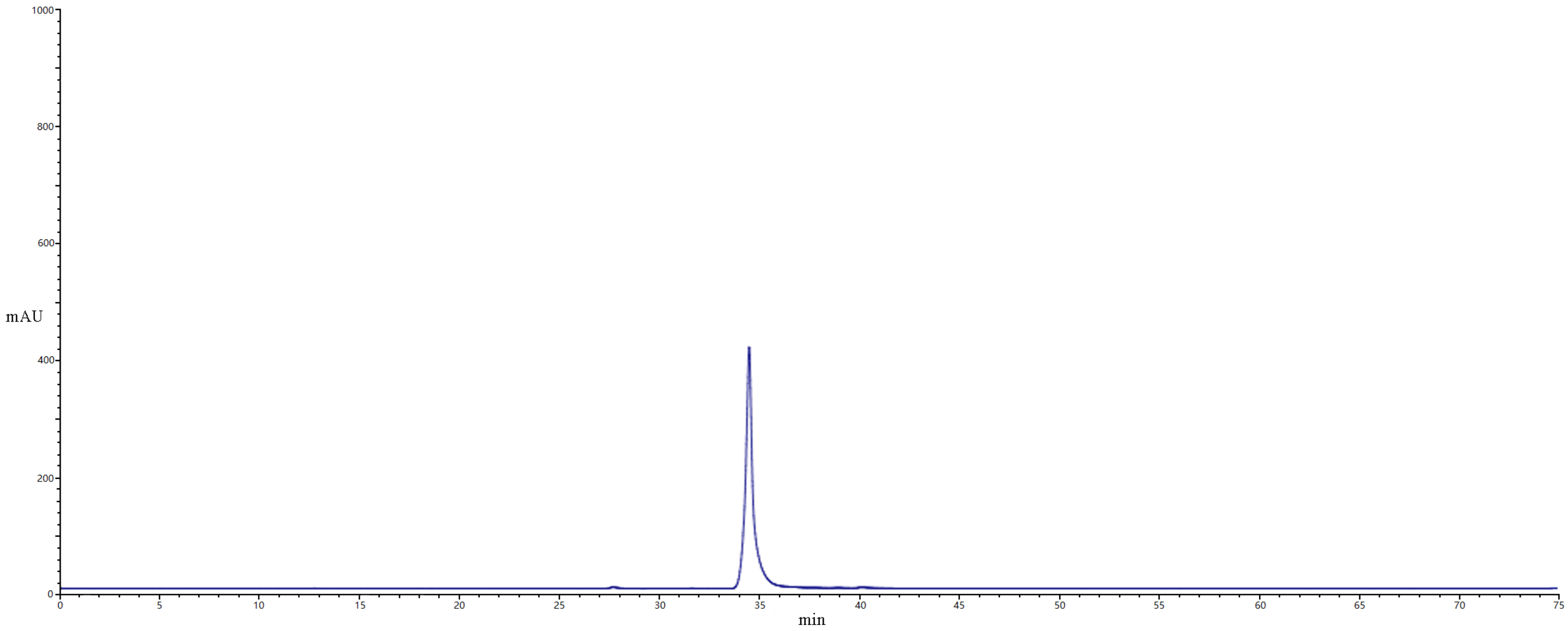

Figure S12: HPLC profile (340 nm) of 5-hydroxy-6,7,3',4'-tetramethoxyflavone (**12**) obtained from the navel orange peel.

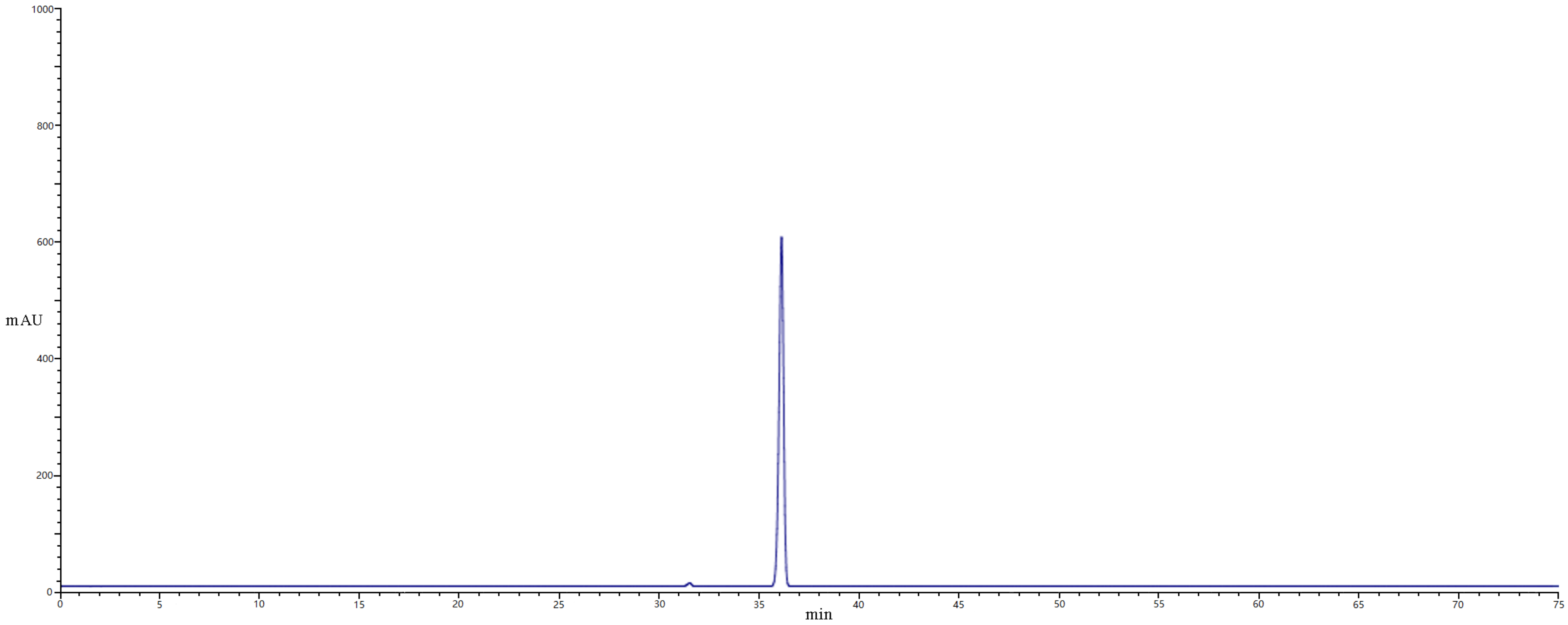

Figure S13: HPLC profile (340 nm) of tangeretin (**13**) obtained from the navel orange peel .
